# Improving text mining in plant health domain with GAN and/or pre-trained language model

**DOI:** 10.3389/frai.2023.1072329

**Published:** 2023-02-21

**Authors:** Shufan Jiang, Stéphane Cormier, Rafael Angarita, Francis Rousseaux

**Affiliations:** ^1^CReSTIC, Université de Reims Champagne Ardenne, Reims, France; ^2^LISITE, Institut Supérieur d'Electronique de Paris, Paris, France

**Keywords:** GAN, social media, plant health monitoring, text classification, pre-trained BERT

## Abstract

The Bidirectional Encoder Representations from Transformers (BERT) architecture offers a cutting-edge approach to Natural Language Processing. It involves two steps: 1) pre-training a language model to extract contextualized features and 2) fine-tuning for specific downstream tasks. Although pre-trained language models (PLMs) have been successful in various text-mining applications, challenges remain, particularly in areas with limited labeled data such as plant health hazard detection from individuals' observations. To address this challenge, we propose to combine GAN-BERT, a model that extends the fine-tuning process with unlabeled data through a Generative Adversarial Network (GAN), with ChouBERT, a domain-specific PLM. Our results show that GAN-BERT outperforms traditional fine-tuning in multiple text classification tasks. In this paper, we examine the impact of further pre-training on the GAN-BERT model. We experiment with different hyper parameters to determine the best combination of models and fine-tuning parameters. Our findings suggest that the combination of GAN and ChouBERT can enhance the generalizability of the text classifier but may also lead to increased instability during training. Finally, we provide recommendations to mitigate these instabilities.

## 1. Introduction

Climate change is causing massive yield losses due to the disruption of cycles and the emergence of crop-affecting pests and plant diseases (Massod et al., 2022). More pest attacks may occur than that reported earlier, and their population may increase due to warmer temperatures. The CO_2_ level and lower soil humidity can also affect the nature of plant diseases (Mozaffari, 2022). To tackle the emerging risks and increasingly unpredictable hazards acting as a menace to crops and plants, precision agriculture emerges as an alternative–or improvement–to existing agricultural practices. Indeed, researchers have experimented with technological innovations to find solutions to some specific goals, such as predicting the climate for agricultural purposes using simulation models (Hammer et al., 2001), improving the efficiency and effectiveness of grain production using computer vision and Artificial Intelligence (Patrício and Rieder, 2018), studying and evaluating soils with drones (Tripicchio et al., 2015), and collecting real-time data from the fields using sensors following the IoT and cloud computing paradigms (Patil et al., 2012). Although the application of these technological innovations produces important results, we suggest that the current observation data from precision agriculture cannot represent all forms of agricultural environments, especially small farms. Recently, the idea of how to encourage the participation of farmers to share their knowledge and observations is drawing the attention of researchers (Jiménez et al., 2016; Kenny and Regan, 2021). Indeed, new studies show that social media might enable farmers to reveal different aspects of their world and to share their experiences and perspectives among colleagues and non-farming audiences (Riley and Robertson, 2021).

The role of social media such as Twitter in farmer-to-farmer and in farmer-to-rural-profession knowledge exchange is increasing, and it suggests that their use among rural professionals and farmers is evolving with open participation (creating contributions), collaboration (sharing contributions), and fuller engagement (asking questions and providing answers/replies) dominating one-way messaging (new/original contributions) (Phillips et al., 2018). Following the *social sensing* paradigm (Wang et al., 2015), individuals—whether they are farmers or not- have more and more connectivity to information while on the move, at the field level. Each individual can become a broadcaster of information by posting real-time hazard observations in social media. Indeed, Twitter enables farmers to exchange experience with each other, to subscribe to topics of interest using hashtags, and to share real-time information on natural hazards. Compared to paid and specialized applications, information on Twitter, presented in the form of text, image, sound, video, or a mixture of the above, is more accessible to the public but less formalized or structured. More and more farmers get involved in online Twitter communities by adding hashtags on their publications to categorize their tweets and help others find them easily (Defour, 2018). Some hashtags are #AgriChatUK^1^, #FrAgTw^2^, and #Farming365.

Still, the extraction of useful plant health information from social media poses some challenges, including lack of context, irrelevancy, homographs, homophones, homonyms, slangs, and colloquialisms. In an earlier study, we developed ChouBERT (Jiang et al., 2022) to detect farmers' observations from tweets for pest monitoring. ChouBERT takes a pre-trained CamemBERT (Martin et al., 2020) model and further pre-trains it on a plant health domain corpus in French to improve the generalizability of plant health hazards detection on Twitter. Some potential applications of ChouBERT are as follows:

- The annotation and indexing of the key elements of plant health-related events in the text, including named entity recognition, entity linking, and relation extraction.
- Topic modeling for detecting emerging issues from a collection of texts.
- Natural language inference for finding precursors of pest attacks.

In this article, we explored the combination of GAN-BERT (Croce et al., 2020) and further pre-training with ChouBERT. We present a discussion on the results and perspectives of this combination on the text classification task for plant health hazard detection.

## 2. Background

### 2.1. Pre-trained language models

Pre-trained language models (PLMs) are deep neural networks of pre-trained weights to vectorize sequences of words. Such vectorial representations obtain state-of-the-art results on NLP tasks, such as text classification, text clustering, question-answering, and information extraction. PLMs suggest an objective engineering paradigm for NLP: language model pre-training for extracting contextualized features from text and fine-tuning for downstream tasks. BERT (Devlin et al., 2019) is a PLM introduced in 2018 by Google that led to significant improvements in this field. BERT is pre-trained in two stages: first, a self-supervised task where the masked language model (MLM) must retrieve masked words in a text; and second, a supervised task where the model must refind whether a sentence B is the continuation of a sentence A or not (next-sentence prediction, NSP). The pre-training produces in the end 12 stacked encoders which take a sequence of tokens as input and add a special token “[CLS]” at the beginning of the sequence and a “[SEP]” at the end of each sentence, and calculates a fixed-length vector for each token. Each dimension of these vectors represents how much attention that token should pay to the other tokens. For the text classification task, the vector of “[CLS]” represents the whole text. Among the French varieties of BERT, CamemBERT (Martin et al., 2020) is a model based on the same architecture as BERT but trained on a French corpus with MLM only. ChouBERT (Jiang et al., 2022) takes a pre-trained CamemBERT-base checkpoint and further pre-trains it with MLM over a corpus in French in the plant health domain to improve performance in detecting plant health issues from short texts, particularly, from Twitter.

### 2.2. Generative adversarial networks

Generative Adversarial Networks (GANs) (Goodfellow et al., 2014; Wang et al., 2017) are a family of neural networks that can be commonly divided into two antagonistic parts: a generator and a discriminator, which compete during training. The generator aims to mimic real data by transforming noise, while the discriminator aims to determine if the data are real or produced by the generator. The discriminator's classification results then feed the generator's training in turn. The training of GANs is known to suffer from the following failure modes: gradient vanish, mode collapse, and non-convergence. Gradient vanish occurs when the discriminator cannot give enough information to improve the generator. Mode collapse occurs when the generator gets stuck generating only one mode. Non-convergence occurs when the generator tends to overfit to the discriminators instead of reproducing the real data distribution.

**Figure 1:**
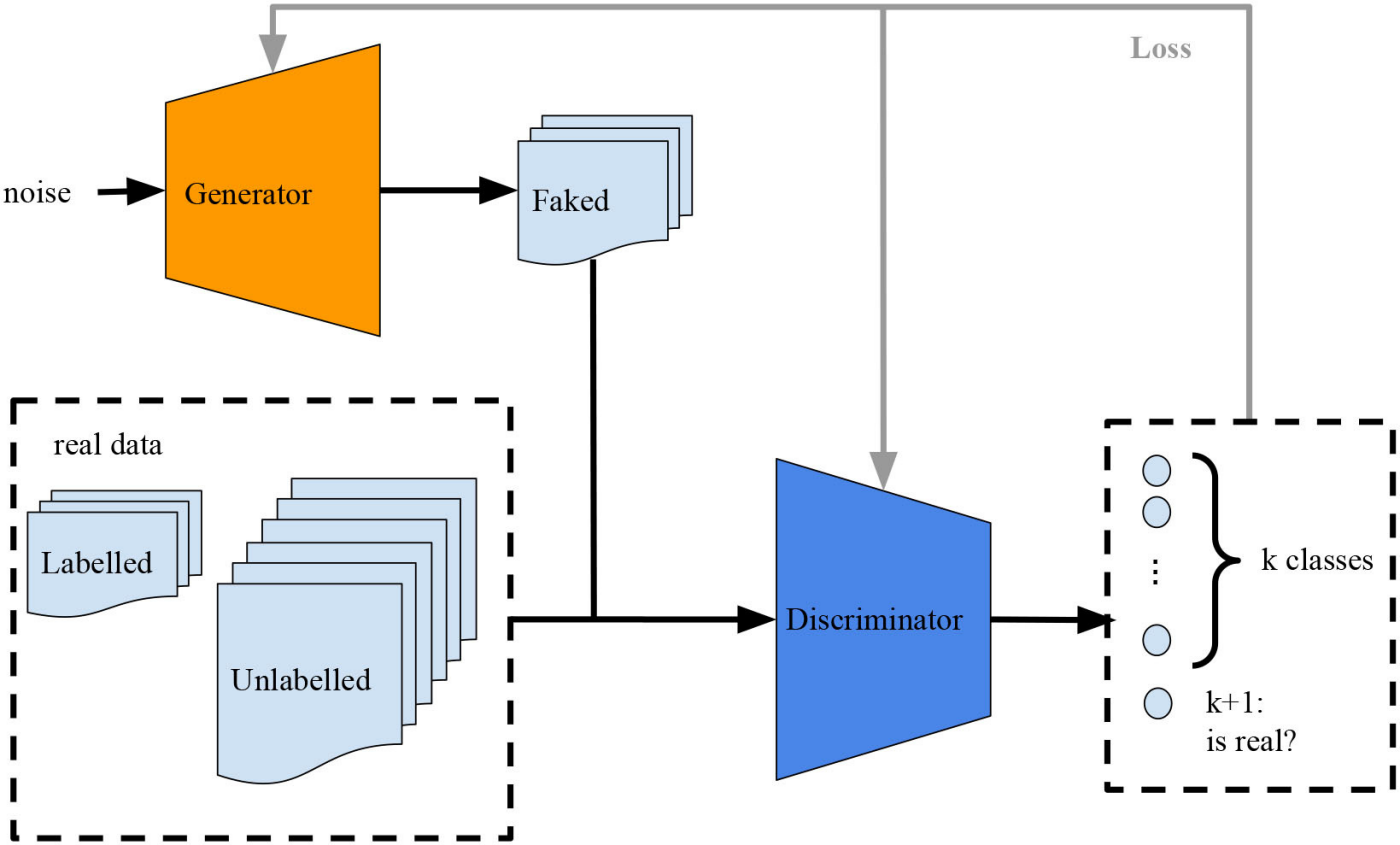
Training an SS-GAN architecture.

Many variants of GANs are proposed to improve sample generation and the stability of training. Some of these variants are the conditional GANs (CGANs), where the generator is conditional on one or more labels (Mirza and Osindero, 2014), and semi-supervised GANs (Salimans et al., 2016) (SS-GANs), where the discriminator is trained over its *k*-labeled examples plus the data generated by the generator as a new label “*k* + 1”(see in Figure 1).

### 2.3. GAN-BERT architecture

Generative adversarial network-bidirectional encoder representations from transformers (GAN-BERT) (Croce et al., 2020) extends the fine-tuning of BERT-like pre-trained language models (PLMs) for text classification with a semi-supervised discriminator–generator setting, introduced in the study by Salimans et al. (2016). Let us project all the data points in a *d*-dimensional hidden space, then the data vector *h* ∈ *R*^*d*^. The generator *G*_*SSGAN*_ is a multi-layer perceptron (MLP) that considers a noise vector as input and attempts to mimic the PLM representation of real data. The discriminator *D*_*SSGAN*_ is another MLP that obtains either PLM representation of real labeled and unlabeled data $h_{R}=PLM\left(x\right),h_{R}\in R^{d}$, or a faked representation $h_{G}=g\left(noise\right),h_{G}\in R^{d}$, produced by *G*_*SSGAN*_ as input, converts the input vector to inner representation $h_{D}\in R^{d}$, and performs a multi-class classification. *D*_*SSGAN*_ is trained over two objectives: 1) the correct classification of real data into *K* classes from labeled data (supervised learning) and 2) the distinction of generated data from real unlabeled data (unsupervised learning).

We define *p*_*m*_(ŷ = *y*|*x, y* ∈ (1, ..., *k*)) as the probability given by the model *m* that an example *x* belongs to one of the *k* target classes and *p*_*m*_(ŷ = *y*|*x, y* = *k* + 1) as the probability of *x* being fake data. Let $P_{R}$ and $P_{G}$ denote the real data distribution and the generated data, respectively. The loss function for training *D*_*SSGAN*_ becomes:


(1) $$\begin{array}{l} L_{D}=L_{D_{sup}}+L_{D_{unsup}} \end{array}$$

*L*_*D*_*sup*__ evaluates how well the real labeled data are classified:


(2) $$\begin{array}{l} L_{D_{sup}}=-{𝔼}_{x,y\sim P_{R}}log\left[p_{m}\left(ŷ\right)=y|x,y\in \left(1,...,k\right)\right] \end{array}$$

*L*_*D*_*unsup*__ punishes the discriminator when it fails to recognize a fake example or when it classifies a real unlabeled example to be fake. If the discriminator is free to assign any of the *k* target classes to the unlabeled data.


(3) $$\begin{array}{l} L_{D_{unsup}}=-{𝔼}_{x\sim P_{R}}log[1-p_{m}(\hat{y}=y|x,y=k+1)]\;\\ \;-{𝔼}_{x\sim P_{G}}log[p_{m}(\hat{y}=y|x,y=k+1)] \end{array}$$

As for the generator *G*_*SSGAN*_, Croce et al. (2020) defines the loss function as:


(4) $$\begin{array}{l} L_{G}=L_{G_{unsup}}+L_{G_{feat}} \end{array}$$

*L*_*G*_*unsup*__ penalizes *G*_*SSGAN*_ when *D*_*SSGAN*_ correctly finds fake examples:


(5) $$\begin{array}{l} L_{G_{unsup}}=-{𝔼}_{x\sim P_{G}}log\left[1-p_{m}\left(ŷ=y|x,y=k+1\right)\right] \end{array}$$

Let *f*_*D*_(*x*) denote the activation that *D*_*SSGAN*_ uses to convert the input data to its inner representation *h*_*D*_. *L*_*G*_*feat*__^3^ measures the statistical distance between the inner representation of real data *h*_*D*_*R*__ and the inner representation of generated data *h*_*D*_*G*__.


(6) $$\begin{array}{l} L_{G_{unsup}}=∥{𝔼}_{x\sim P_{R}}f\left(x\right)-{𝔼}_{x\sim P_{G}}f\left(x\right){∥}_{2}^{2} \end{array}$$

The PLM is part of the discriminator *D*_*SSGAN*_; that is, when updating *D*_*SSGAN*_, the weights of the PLM are also fine-tuned. Moreover, at the beginning of each training epoch, the [CLS] vector of real examples is recalculated by the updated PLM.

### 2.4. GAN-BERT applications

Generative adversarial network-bidirectional encoder representations from transformers (GAN-BERT) has been assessed on different datasets with different PLMs. The original authors of GAN-BERT have applied it to English sentence-level classification tasks, including topic classification, question classification (QC), sentiment analysis, and natural language inference (NLI) with the original BERT model (Devlin et al., 2019; Croce et al., 2020).

Later, MT-GAN-BERT (Breazzano et al., 2021) extends GAN-BERT to a multi-task learning (MTL) architecture to solve simultaneously several related sentence-level classification tasks reducing overfitting. MT-GAN-BERT is assessed with English and Italian datasets, using BERT and UmBERTo^4^, respectively, for sentence embedding generation. The results of MT-GAN-BERT show that GAN-BERT-based models outperform BERT-based models with 100 and 200 labeled data. However, the performance worsens when training GAN-BERT with 500 labeled data.

In the study of Ta et al. (2022), the authors applied GAN-BERT for paraphrase identification. They propose to filter noises in the labeled set to improve the performance and claim that, for their use case, a lower learning rate helps the model to learn better. However, a too-small learning rate makes the accuracy to increase slowly. In the study of Santos et al. (2022), the authors applied GAN-BERT with Portuguese PLMs to find hate speech in social media. This study shows that text cleaning, including removing users' mentions, links, and repeated punctuation, improves the performance of GAN-BERT-based classification. Finally, the authors infer that GAN-BERT is nonetheless more susceptible to noise.

In the study of Myszewski et al. (2022), the authors showed that the combination of a GAN-BERT setting with a domain-specific PLM BioBERT (Lee et al., 2019) outperforms the original GAN-BERT on a sentiment classification task for clinical trial abstracts. However, the authors do not compare the results with those of PLM-only classification. They neither provide a detailed analysis of the training. In this study, the authors presented 108 labeled examples. The small number (23) of labeled samples in their test set also makes the result unconvincing, which calls for more studies to validate the combination of GAN-BERT and domain-specific PLMs.

In the study of Danielsson et al. (2022), the authors studied whether and how GAN-BERT can help in the classification of patients bearing implants with a relatively small set of labeled electronic medical records (EMRs) written in Swedish. In practice, they further pre-trained a Swedish BERT model^5^ to provide the [CLS] representations of 64 and 512 tokens to the discriminator of GAN-BERT and perform experiments over varying training set sizes. Their results show that combining GAN-BERT and a domain-specific PLM improves the classification performance in specific challenging scenarios. However, the effective zone of such scenarios remains to be studied. The numerous applications of GAN-BERT witness its capacity for fine-tuning PLM on sentence-level classification tasks in a low resource setting. However, none of the works presented in this section have studied the correlation between the labeled/unlabeled data ratio and the performance of GAN-BERT or the impact of using domain-specific PLMs. The lack of specifications for these hyperparameters makes the GAN-BERT setting a black box to newcomers and could lead to expensive grid search experiments for optimization (Bergstra and Bengio, 2012). Furthermore, the granularities of different classification problems are not comparable. Therefore, it is unfair to compare the performances of GAN-BERT plus the PLMs pre-trained in different languages or domains over these tasks. In this study, we address these shortcomings by applying the GAN-BERT settings to CamemBERT (Martin et al., 2020), ChouBERT-16, and ChouBERT-32 and probing the different losses over varying labeled and unlabeled data sizes to give more insights into when and how to train GAN-BERT for domain-specific document classification.

## 3. Method

### 3.1. Data

Data annotation by domain experts is expensive and time-consuming. Therefore, the main challenge of detecting natural hazards from textual contents on social media is to identify unseen risks with low resources for training. We reuse the labeled tweets produced by ChouBERT (Jiang et al., 2022), tweets about corn borer, barley yellow dwarf virus (BYDV) and corvids for training and validation, and tweets about unseen and polysemous terms such as “taupin” (wireworm in English) for testing the generalizability of the classifier. Since the binary cross entropy loss adopted by the discriminator of GAN-BERT favors the majority class when data are unbalanced, for the different training experiments, we sampled ChouBERT's training data to 16, 32, 64, 128, 256, and 512 subsets, each subset having equal number of observations and non-observations. We used the same validation data and test sets for all the experiments. In the validation set, there were 79 observations and 213 non-observations; in the test set, there were 58 observations and 447 non-observations.

Among the data collected by ChouBERT, there is not only a small set of labeled tweets but also many unlabeled tweets. For the unsupervised learning, we have 12,308 unlabeled tweets containing common insect pest names (other than those in the labeled data) in France. We sampled 0, 1,024, 4,096, and 8,192 unlabeled data to study the effect of adding unlabeled data.

### 3.2. Text classification with a pre-trained language model

Following the study of Jiang et al. (2022), the ChouBERT models^6^ are further-pre-trained CamemBERT-base models over French Plant Health Bulletins and Tweets and the ChouBERT pre-trained for 16 epochs (denoted as ChouBERT-16) and for 32 epochs (denoted as ChouBERT-32) are the most efficient in finding observations about plant health issues. Thus, in this study, we combine GAN-BERT settings with CamemBERT, ChouBERT-16, and ChouBERT-32.

To make our state-of-the-art model, we fine-tune CamemBERT, ChouBERT-16, and ChouBERT-32 for the sequence classification task over the same training/validation/test sets by adding a linear regression layer *a* to the final hidden state *h* of the [CLS] token to predict the probability of a label *o*:


(7) $$\begin{array}{l} p\left(o|h\right)=softmax\left(W_{a}h\right), \end{array}$$

Where *W*_*a*_ is the parameter matrix of this linear classifier.

During the training, the weights of the PLM are affected along with *W*_*a*_. We developed these experiments with *CamemBertForSequenceClassification* of the transformer package^7^. To make the probability outputs of this linear regression layer comparable with the label outputs from the GAN-BERT classifier, we fixed the threshold of 0.5. For the predicted probability greater than 0.5, we considered it as an observation, else as a non-observation. It is worth mentioning that we used 0.5 as a threshold to simplify the comparison with the PLM plus GAN-BERT classification. When applying the PLM-only classification to other datasets in other domains, we might need to find an optimal threshold depending on the real needs for precision or recall. Based on the results presented in the study of Jiang et al. (2022), we fixed the learning rate to 2e^−5^, the maximum sequence length to 128, and fit the classifier for 10 epochs. We set the batch size to (*training*_*data*_*size*/8) to have the same steps for the different training data sizes.

### 3.3. Experimental setup

For our experiments, we used GAN-BERT's latest PyTorch implementation,^8^ which is compatible with the transformer package. We fixed the max sequence length of the PLM to 128. We fixed the number of hidden layers in *G* and in *D* to 1 and the size of *G*'s input noisy vectors to 100. We used the following learning rate combinations (*D, G*): (5e-5, 5e-5), (1e-5, 1e-5), and (5e-6, 1e-6). We applied the AdamW (Loshchilov and Hutter, 2017) optimizer with and without a cosine scheduler. To limit the number of variables, we conduct two groups of experiments. In the first group, we fixed the batch size per GPU to 32 and epochs to 30. We trained the GAN-BERT architectures over increasing labeled data sizes (16, 32, 64, 128, 256, and 512) and unlabeled data sizes (1,024, 4,096, and 8,192). In the second group, we fixed the training steps of each (labeled and unlabeled) pair by setting the batch size to (*unlabeled*_*data*_*size*/256). Moreover in the second group, we trained the GAN without unlabeled data. That is, in this group, the unsupervised learning learns the features from labeled data only. We fixed the batch size to 4 and set epochs to (1, 024/*train*_*data*_*size*+*log*_2_(*train*_*data*_*size*)) to approximate the number of training steps in the experiments with unlabeled data.

## 4. Results and evaluation

### 4.1. Overall metrics

As the validation set and the test set were unbalanced and that our interest is to find out the observations, we plot the F1 score of the observation class *F*1_*observation*_ and the macro average F1 score of the whole classification.


(8) $$\begin{array}{l} F1_{macro}=\left(F1_{observation}+F1_{non-observation}\right)/2 \end{array}$$

Let us consider a dummy classifier as our baseline model. If it predicts that all the examples in the validation set are non-observations, the *F*1_*observation*_, *F*1_*macro*_, and accuracy become 0, 0.42, and 0.73, respectively; if it predicts that all are observations, the *F*1_*observation*_, *F*1_*macro*_, and accuracy become 0.43, 0.21, and 0, respectively.

**Figure 2:**
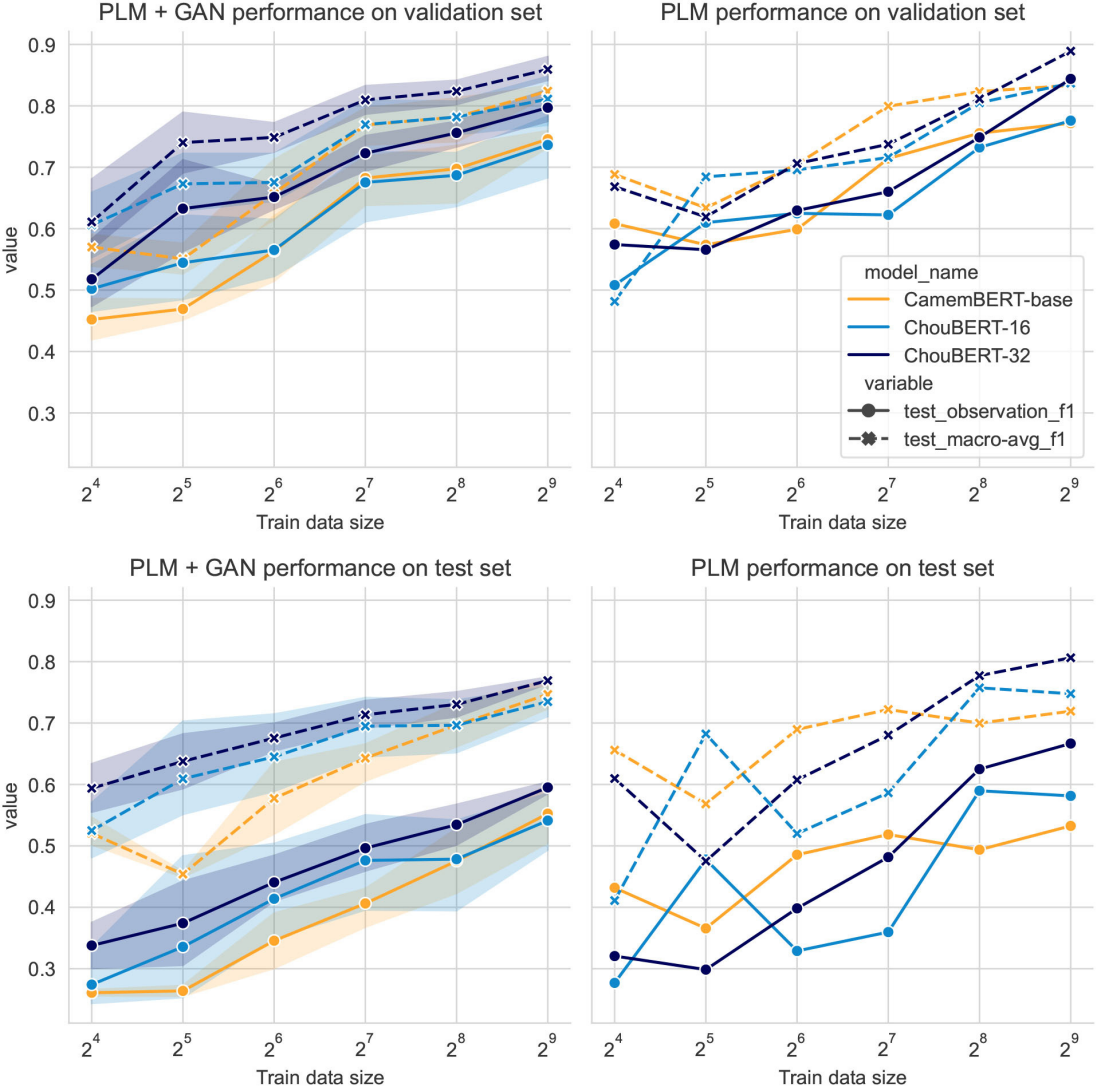
PLM + GAN-BERT vs. PLM only with same training steps over varying sizes of training datasets.

**Figure 3:**
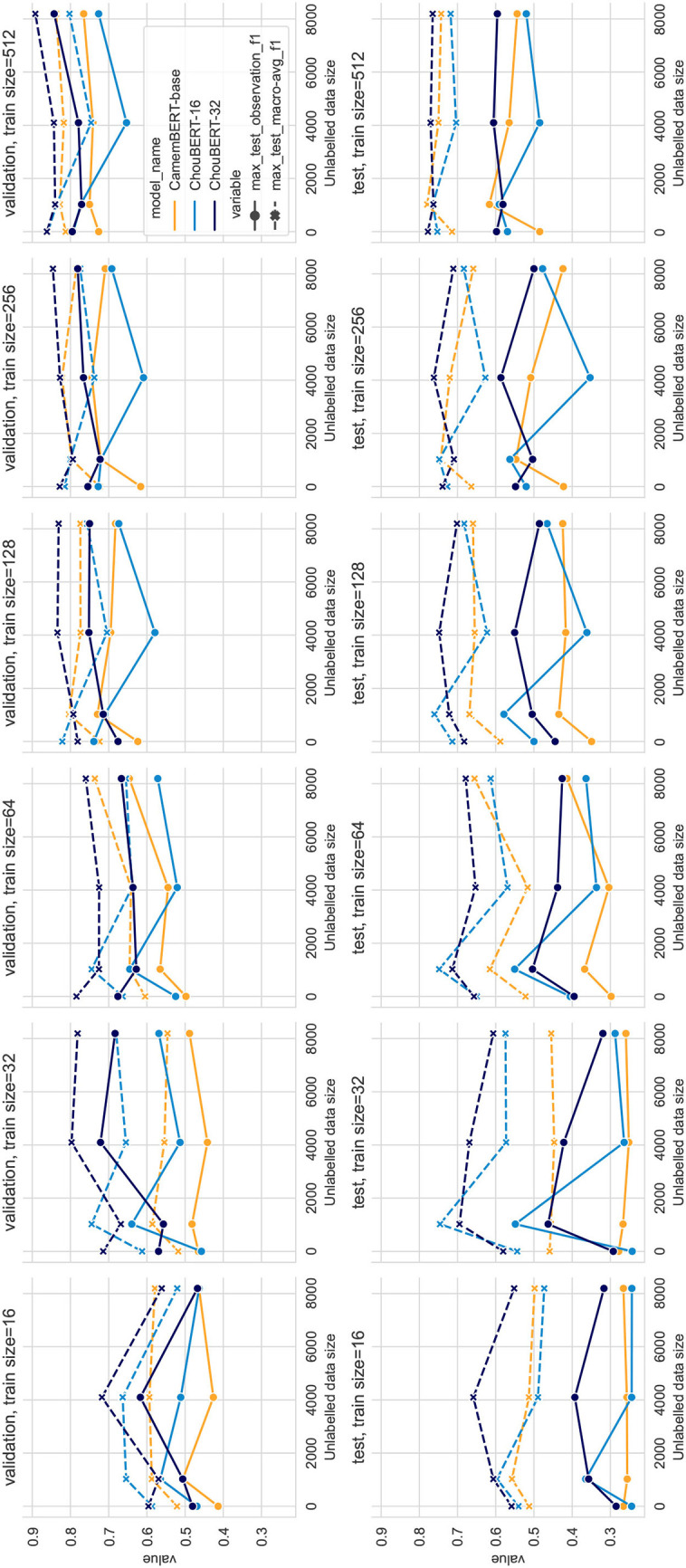
PLM + GAN-BERT performance with fixed steps, training dataset sizes = 16, 32, 64, 128, 256, and 512.

**Figure 4:**
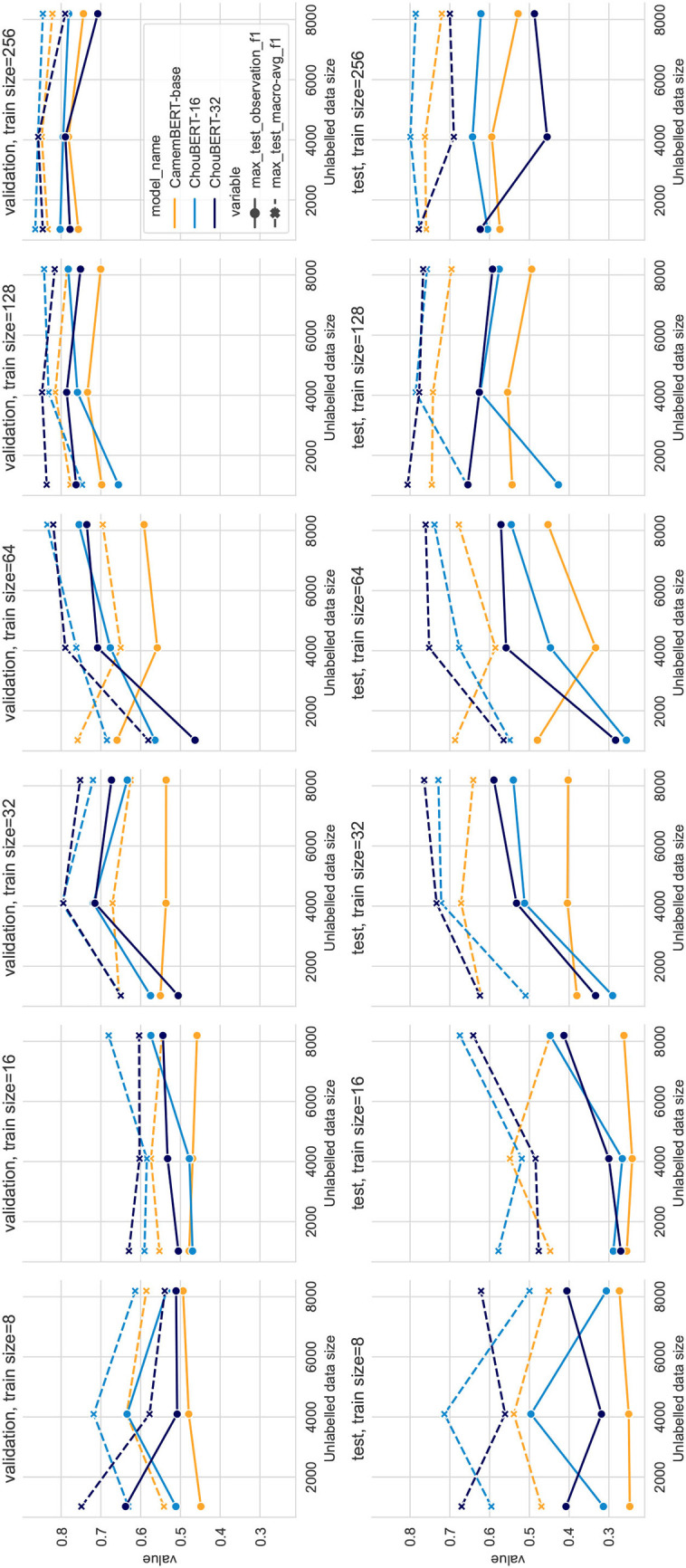
PLM + GAN-BERT performance with fixed batch size to 32, training dataset sizes = 16, 32, 64, 128, 256, and 512.

We present the overall results of the fixed-step experiments in Figure 2, which are the most representative and stable. By comparing the maximum F1 scores of each configuration during the training in Figures 2–**4**, we believe the performance of the classifiers on both the validation and test sets to be continuous and relatively stable in a period, once the training converges. In other words, overfitting will not immediately cause huge drops. It is worth pointing out that, on the unbalanced validation and test sets, the F1 score (the objective of our classification task) and the binary cross entropy loss (the objective of GAN-BERT's training) are not completely aligned and may lead to suboptimal convergence.

Compared to PLM-only classification, the PLM plus GAN-BERT setting improves the scores over the validation and test sets of unseen hazards with 32, 64, 128, and 256 training data. In Figure 3, we depict the performance with varying unlabeled data sizes. In both figures, we can see that the deep blue lines (ChouBERT-32) are above the yellow lines (CamemBERT), which is clearly coherent with the results presented in Jiang et al. (2022), indicating that pre-training helps to improve generalizability. The representational similarity analysis in Merchant et al. (2020) shows that “fine-tuning has a much greater impact on the token representations of in-domain data” and suggests fine-tuning to be “conservative.” In our experiments, we did not observe that the SSGAN setting without domain unlabeled data helps the model generalization for the identification of tweets about upcoming unseen hazards. For small training data sizes, adding unlabeled data helps to improve the performance on the test set, but adding more unlabeled data consumes more computational resources without making significant difference. We observe similar phenomena in the fixed batch size group results in Figure 4, where adding more unlabeled data brings more training steps per epoch and eventually reduces the *L*_*sup*_ steadily within the same training epochs.

In all our experiments with 512 labeled data, PLM-only solutions outperform PLM plus GAN-BERT solutions, while, in experiments, with between 32 and 256 labeled data, PLM+GAN-BERT improves the performance on the validation and test sets, which corresponds to the results presented in Breazzano et al. (2021) and Danielsson et al. (2022).

### 4.2. The instability of the GAN-BERT setting with ChouBERT models

**Figure 5:**
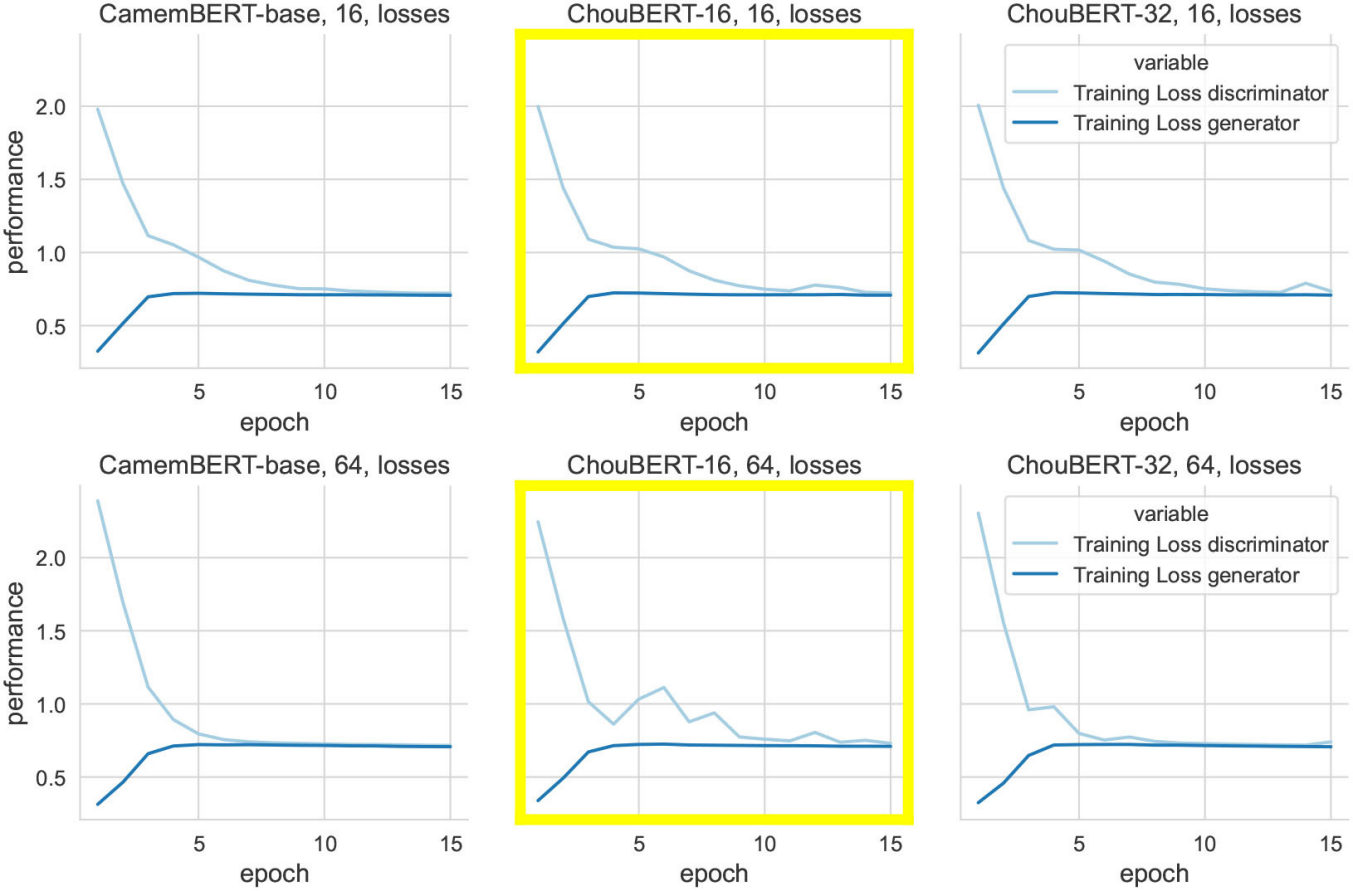
A macroscopic view of the evolution of training losses, with labeled data sizes of 16 and 64 over 15 epochs. We fix unlabeled size to 4096, Learning rates of the Discriminator and Generator to 5e-6 and 1e-6.

**Figure 6:**
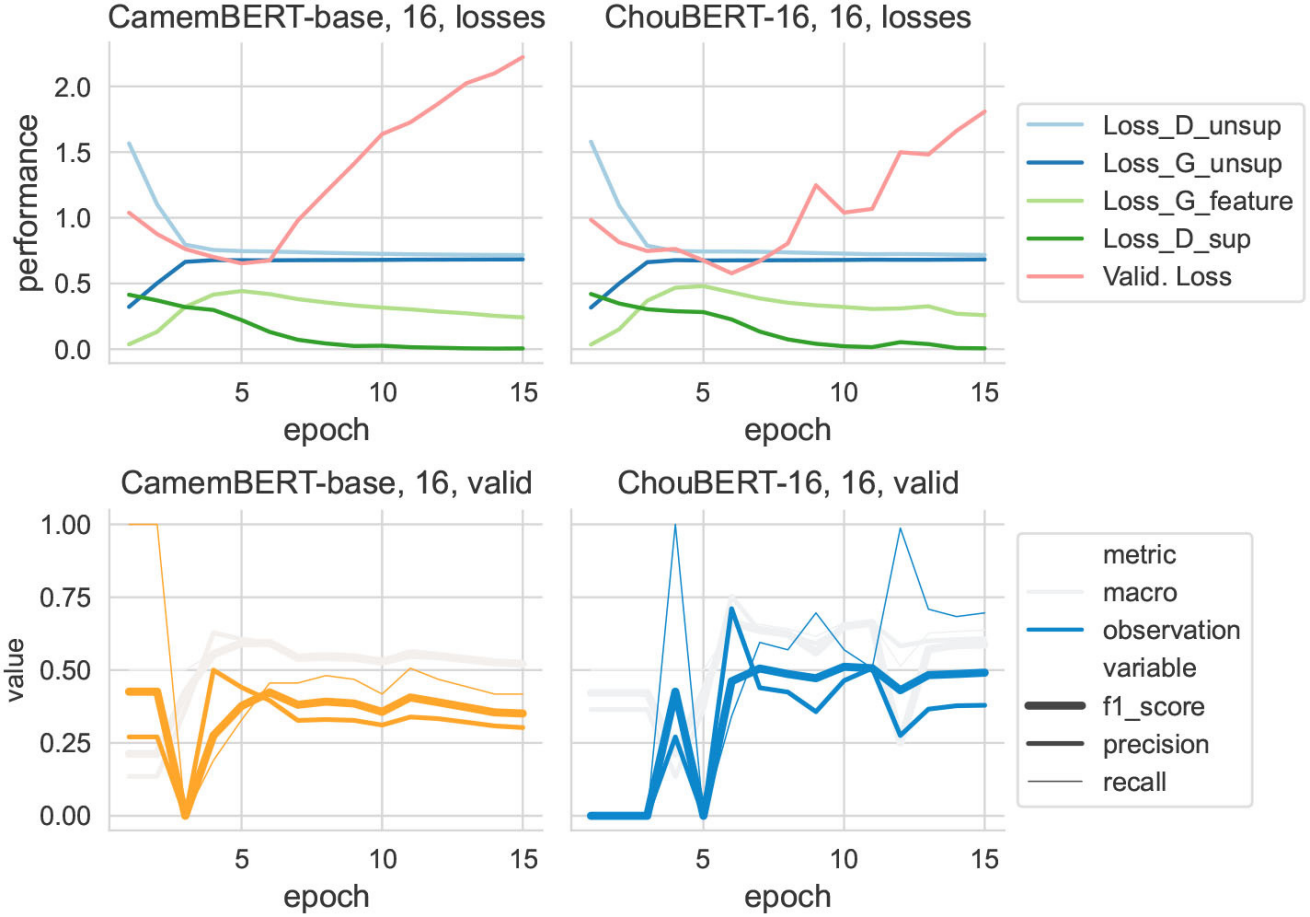
Training data set size = 16.

**Figure 7:**
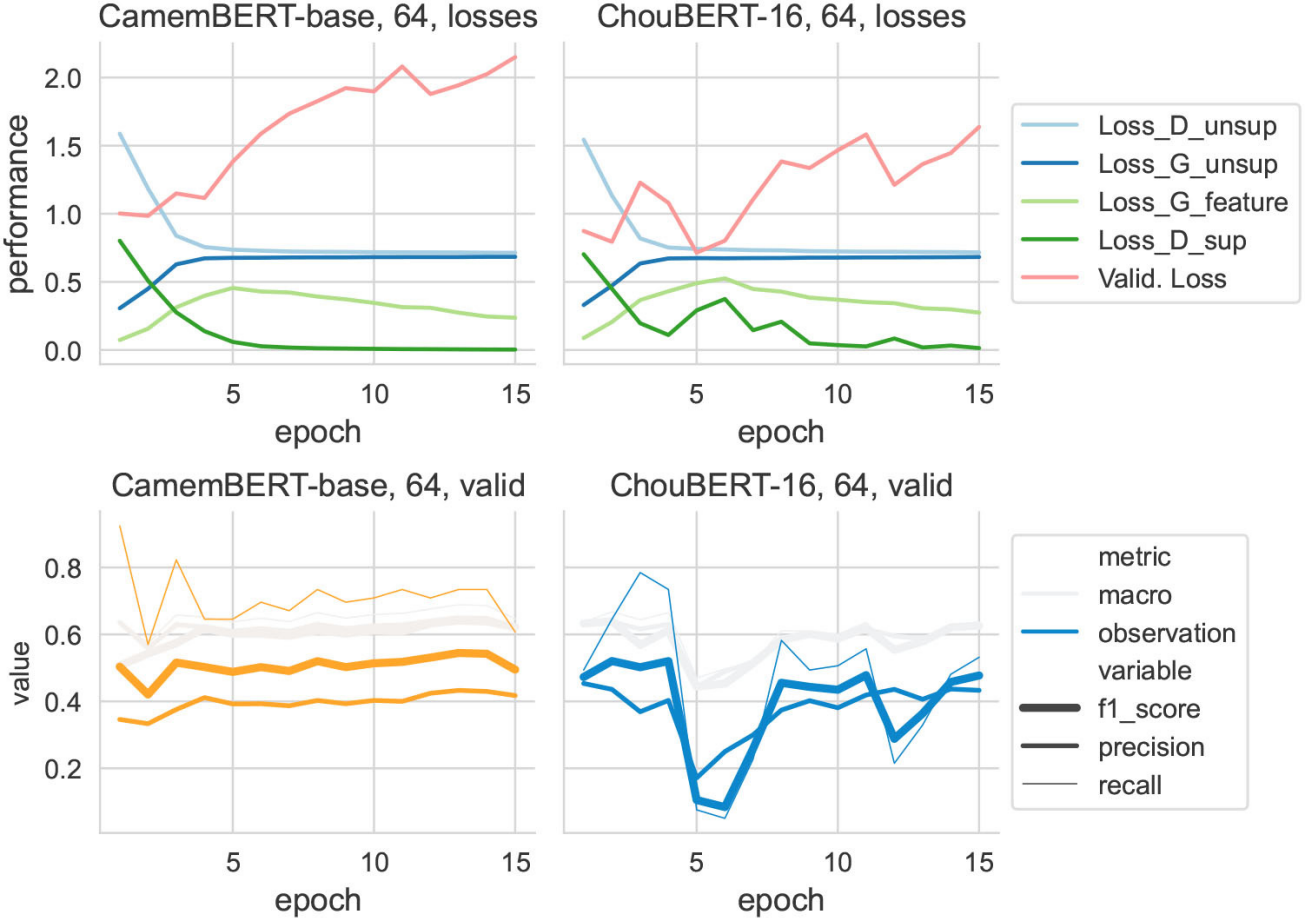
Training data set size = 64.

Even though the fine-tuning of pre-trained transformer-based language models such as BERT has achieved state-of-the-art results on NLP tasks, fine-tuning is still an unstable process. Training the same model with multiple random seeds can result in different performances on a task as described in the study of Mosbach et al. (2021). This training instability is the reason why we have not found the best labeled/unlabeled ratio to maximize the performance. In Figure 5, we illustrate the training losses of the discriminator and the generator when given different sizes of labeled data with fixed unlabeled data size and learning rate. The training with ChouBERT models has more difficulties to converge than with CamemBERT. Thus, we explored the evolution of different losses and the classifiers' performance metrics on the validation and test sets in Figures 6, 7, where the discriminators' losses with ChouBERT-16 take more epochs to decrease than with CamemBERT.

It is immediately clear that discriminators' losses had the same shape as *L*_*sup*_. In particular, to present the evolution of *L*_*G*_*feat*__ at the same scale as the other losses, we multiplied its value by 10 to draw its line. Compared with ChouBERT-16, the *L*_*sup*_ had more difficulties decreasing than with CamemBERT. We interpret the increase of *L*_*G*_*feat*__ as the generator catching up with the fine-tuning of PLM and the decrease of *L*_*G*_*feat*__ toward its initial value as the end of the major changes of fine-tuning.

According to the authors of SSGAN (Salimans et al., 2016), “in practice, *L*_*unsup*_ will only help if it is not trivial to minimize for our classifier and we thus need to train G to approximate the data distribution,” which explains that, while the *L*_*unsup*_ of *D* and *G* converge at the same rhythm with CamemBERT and with ChouBERT-16, the troubled decrease of *L*_*sup*_ with ChouBERT-16 renders worse F1 scores than those with CamemBERT. For example, in the group with 16 training examples (see Figure 6), the test *F*1_*observation*_ scores with ChouBERT-16 are switching between 0 and 0.43, which means that the classifier predicts either all as non-observation or all as observation. Considering the unbalanced nature of our validation and test sets, all-observation predictions and all-non-observation predictions are two local Nash equilibria for the training of our SSGAN.

It is also remarkable that, in the group with 64 training examples (see Figure 7), ChouBERT-16 gives better F1 scores than CamemBERT in the early stages. However, after the bounces of *L*_*sup*_, despite the fine-tuning, helps it to decrease again, the F1 scores are not as good as before because the effect of *L*_*unsup*_ is already gone. We can also observe this phenomenon in the group with 32 training examples. Interestingly, when repeating the experiments with the same hyperparameters, the “troubled decrease” of *L*_*sup*_ does not always happen, but statistically, most of them can happen with ChouBERT models, especially ChouBERT-16. Our strategies against the “troubled decrease” include the following:

- Using a smaller learning rate with more training epochs at the cost of computational resources (see Ta et al., 2022).
- Applying a smaller learning rate to *G* than to *D* (see Heusel et al., 2017).
- Applying schedulers and down-sampling the majority class to balance the training data–in our case, the upsampling proposed by the original code of GAN-BERT does not help.

With the optimizations mentioned above, *L*_*sup*_ with CamemBERT decreases at a steady pace and “troubled decrease” happens less often with ChouBERT models. When we examined the embeddings of [CLS] produced by the PLM, we found that there is more variance in each dimension of CamemBERT embeddings than in each dimension of ChouBERT embeddings, before and after the fine-tuning: *Var*_*CamemBERT*_ > *Var*_*ChouBERT*−32_ > *Var*_*ChouBERT*−16_. Thus, ChouBERT models produce more homogeneous encodings than CamemBERT. This explains why ChouBERT embeddings are more generalizable for detecting unseen hazards: the embeddings of texts containing unseen hazards are more similar to those of seen hazards, so the downstream classifier is more familiar with these vectors. It also indicates that the differences between observations and non-observations are more subtle in ChouBERT's latent space. Thus, the training of GAN plus ChouBERT needs lower learning rates to converge, while GAN plus CamemBERT is a robust approach to converge in most configurations.

## 5. Conclusion

In this article, we demonstrate that combining further-pre-trained language models and GAN-BERT benefits from the generalizability of the domain-specific PLM to classify unseen hazards. We also demonstrate that training such a combination may also suffer from extra instabilities compared to using GAN-BERT with CamemBERT, a general PLM. Our results validate that the GAN-BERT setting improves the task of natural hazard classification for datasets containing between 32 and 256 instances of labeled data.

Based on our experimental studies, we give our suggestions to reduce the instability such as: (1) The *L*_*sup*_ needs a certain minimum number of steps to decrease to zero. For a fixed batch size, adding unlabeled data makes more training steps to go through in each epoch, consequently allowing *L*_*sup*_ to decrease at a similar pace as *L*_*unsup*_. When the number of unlabeled data is limited, using smaller batch sizes and training for more epochs is also a good approach. (2) If the task is not too domain-specific, in other words, when the further pre-trained language model cannot significantly outperform the general language model in the PLM-only classification, using a general language model with the GAN-BERT setting is safer. On the other hand, if the task is highly domain-specific, it is better to apply schedulers, downsample the majority class to balance the training data, and use smaller learning rates to train GAN-BERT with further-pre-trained language models. (3) We need to choose a suitable PLM. We proved that ChouBERT-32 outperforms ChouBERT-16 in an SSGAN setting. The perspectives and developments are numerous to increase the stability of domain-specific text classification using GAN-BERT; for example, how to further pre-train PLMs to adapt better SSGAN setting is yet to investigate.

## Data availability statement

The data analysed in this study are subject to the following licenses/restrictions: Redistribution of the collected Twitter data is restricted by the Twitter Terms of Service, Privacy Policy, Developer Agreement, and Developer Policy. The tweet IDs of the labeled tweets and the labels will be made available by the authors.

## Author contributions

SJ and SC were responsible for the conception. RA, SC, and FR were responsible for administrative support and the provision of study materials. SJ was responsible for data collection and experiments. All authors contributed to the manuscript revision, read, and approved the submitted version.

## Footnotes

^1^ http://www.agrichatuk.org

^2^ https://franceagritwittos.com

^3^In the PyTorch implementation of the feature reg loss, the authors use: g_feat_reg = torch.mean(torch.pow(torch.mean(D_real_features, dim=0) - torch.mean(D_fake_features, dim=0), 2)), which is not exactly the same as the definition given by the article; however, according to other author's experiments and our experience, there is no significant impact on the training result.

^4^ https://huggingface.co/Musixmatch/umberto-wikipedia-uncased-v1

^5^ https://github.com/Kungbib/swedish-bert-models

^6^ https://huggingface.co/ChouBERT

^7^ https://huggingface.co/transformers/v3.0.2/

^8^ https://github.com/crux82/ganbert-pytorch
